# Rapamycin-Loaded Lipid Nanocapsules Induce Selective Inhibition of the mTORC1-Signaling Pathway in Glioblastoma Cells

**DOI:** 10.3389/fbioe.2020.602998

**Published:** 2021-02-25

**Authors:** Delphine Séhédic, Loris Roncali, Amel Djoudi, Nela Buchtova, Sylvie Avril, Michel Chérel, Frank Boury, Franck Lacoeuille, François Hindré, Emmanuel Garcion

**Affiliations:** ^1^Univ Angers, Université de Nantes, Inserm, CRCINA, SFR ICAT, Angers, France; ^2^Université de Nantes, Inserm, CNRS, CRCINA, Nantes, France

**Keywords:** rapamycin, nanoparticles, radiation, hypoxia, mTOR, Akt, HIF-1α, cancer

## Abstract

Inhibition of the PI3K/Akt/mTOR signaling pathway represents a potential issue for the treatment of cancer, including glioblastoma. As such, rapamycin that inhibits the mechanistic target of rapamycin (mTOR), the downstream effector of this signaling pathway, is of great interest. However, clinical development of rapamycin has floundered due to the lack of a suitable formulation of delivery systems. In the present study, a novel method for the formulation of safe rapamycin nanocarriers is investigated. A phase inversion process was adapted to prepare lipid nanocapsules (LNCs) loaded with the lipophilic and temperature sensitive rapamycin. Rapamycin-loaded LNCs (LNC-rapa) are ~110 nm in diameter with a low polydispersity index (<0.05) and the zeta potential of about −5 mV. The encapsulation efficiency, determined by spectrophotometry conjugated with filtration/exclusion, was found to be about 69%, which represents 0.6 wt% of loading capacity. Western blot analysis showed that LNC-rapa do not act synergistically with X-ray beam radiation in U87MG glioblastoma model *in vitro*. Nevertheless, it demonstrated the selective inhibition of the phosphorylation of mTORC1 signaling pathway on Ser2448 at a concentration of 1 μM rapamycin in serum-free medium. Interestingly, cells cultivated in normoxia (21% O_2_) seem to be more sensitive to mTOR inhibition by rapamycin than those cultivated in hypoxia (0.4% O_2_). Finally, we also established that mTOR phosphorylation inhibition by LNC-rapa induced a negative feedback through the activation of Akt phosphorylation. This phenomenon was more noticeable after stabilization of HIF-1α in hypoxia.

## Introduction

Glioblastoma (GB) is the most common and deadly primary brain tumor in adults (Ostrom et al., 2017). Despite remarkable advances in surgical techniques and treatment options including chemotherapy and radiotherapy, the prognosis of this disease remains very poor with a median survival under 15 months (Stupp et al., 2005, 2009). Therefore, the understanding of the molecular mechanisms that drive malignancy in glioblastoma is seriously needed for the development of new agents specifically targeting tumor cells and the tumor microenvironment (Touat et al., 2017; Najberg et al., 2019).

The phosphatidylinositol 3-kinase (PI3K)/protein kinase B (Akt)/mechanistic target of rapamycin (mTOR) intracellular signaling pathway plays a central role in the regulation of cell proliferation, growth, differentiation, and survival (Sonoda et al., 2001; Bjornsti and Houghton, 2004; Knobbe et al., 2005; Castellino and Durden, 2007; Jiang and Liu, 2009). Stimulation of this pathway results in the activation of a receptor tyrosin kinase (RTK) by a cytokine or a growth factor, which drive a sequential phosphorylation of PI3K, Akt, and mTOR. mTOR regulates cell growth and survival *via* two different multiprotein complexes, mTORC1 and mTORC2. The complex mTORC1 is composed of mTOR, regulatory-associated protein of mTOR (Raptor), mammalian lethal with Sec13 protein 8 (mLST8), proline-rich AKT substrate 40 kDa (PRAS40), and DEP-domain-containing mTOR-interacting protein (Deptor) (Saxton and Sabatini, 2017). mTORC1 activates the eukaryotic initiation factor 4E (eIF4E)-binding protein, releasing the transcription factor eIF4E and the p70 ribosomal S6 kinase 1 (S6K1 or p70S6K) implicated in translation (Heimberger et al., 2005).

This pathway can be activated through numbers of mechanisms, including growth factors, overexpression or amplification of Akt family members, inactivation of the inhibitory effects of PTEN (phosphatase and tensin homolog) tumor suppressor or by non-canonical Wnt pathway (Saxton and Sabatini, 2017). Furthermore, radiation can also activate mTOR signaling in vascular endothelium and in glioblastoma cell lines (Eshleman et al., 2002; Shinohara et al., 2005; Anandharaj et al., 2011). Consequently, mutations in the PI3K or AKT genes, loss of PTEN, epigenetic modifications, or constitutive activation of upstream tyrosine kinase receptors will lead to dysregulation of this pathway in a variety of tumors, including GB (Engelman, 2009; Bai et al., 2011; Wick et al., 2011; Mao et al., 2012). As such, there are marked associations between alterations in the PI3K/AKT/mTOR pathway and the poor clinical survival (Engelman, 2009). Therefore, inhibition of the PI3K/Akt/mTOR signaling pathway has been widely investigated as a potential therapy for cancer including glioblastoma (Li et al., 2016). Interestingly, tumor cells in which the PI3K/Akt/mTOR pathway is dysregulated are more susceptible to the inhibition of mTOR, the downstream effector of this signaling pathway, than normal cells (Courtney et al., 2010). Hence, mTOR inhibitors such as rapamycin and its derivatives provide a new class of active agents and therapeutics for GB.

Rapamycin (Sirolimus) is a natural macrolide antibiotic (firstly isolated from samples of Streptomyces hygroscopicus found on Easter Island), which binds to FK506 binding protein 12 (FKBP12). The rapamycin-FKBP12 complex inhibits mTOR and prevents further phosphorylation of proteins involved in the transcription, translation, and cell cycle control (Heimberger et al., 2005). Anandharaj et al. studied three PTEN-null GB cell lines and demonstrated that rapamycin combined with radiotherapy inhibited the inhibitor of apoptosis protein (IAP) family protein surviving through repression of phospho-Akt. Thus, targeting Akt through mTOR with rapamycin increased the radiation sensitivity (Anandharaj et al., 2011). Preclinical trials showed that PTEN deficient tumors and those dependent on PI3K overexpression were most sensitive to rapamycin (Bjornsti and Houghton, 2004). These results provide a strong basis for investigation of mTOR inhibitors as potential tumor-selective therapeutic agents. Rapamycin and its derivatives, CCI-779 and RAD001, specifically inhibit the function of mTOR by blocking the phosphorylation of downstream molecules, such as p70S6 kinase (p70S6K) and eukaryotic initiation factor 4E-binding protein 1 (4E-BP1), leading to G1-phase cell cycle arrest. Accumulating evidence from preclinical and early clinical studies suggests that these mTOR inhibitors, alone or in combination, would be directly and indirectly effective as growth inhibitors against a broad range of tumors including GB (Mecca et al., 2018; Hsu et al., 2020; Wanigasooriya et al., 2020).

Despite the potency of rapamycin in preclinical studies, clinical development of rapamycin floundered due to the lack of suitable formulations. The low oral bioavailability (<15%) (Yatscoff et al., 1995) precludes tablet formulation except for low dosage treatments such as immunosuppression. Rapamycin's poor solubility in water, ca. 2.6 μg/mL, and common excipients make intravenous (i.v.) formulation difficult (Simamora et al., 2001). In addition, pharmacokinetic studies found that rapamycin strongly partition into the erythrocytes (Kd ca. 20) from where it may not readily access to solid tumors (Yatscoff et al., 1995). This led to the development of ester derivatives, e.g., Temsirolimus or CCI-779, which were more easily formulated. Despite the promise of CCI-779 for mTOR inhibition, intravenous formulations required ethanol that may cause hemolysis (Raymond et al., 2004). Furthermore, phase I trials established that the CCI-779 prodrug was rapidly hydrolyzed in the plasma back into rapamycin thus favoring again potential partition into erythrocytes and unsupportive for tumor accumulation. More recent evolutions with the derivative Everolimus in phase II leads to increase treatment-related toxicities (Chinnaiyan et al., 2018).

In order to improve rapamycine biodistribution, nanovectorization strategies have been developed. They provide a physical protection and allow freeing from solubility problems. In this work, lipid nanocapsules loaded with rapamycin (LNC-rapa) were developed as new nanocarriers for the treatment of GB. We demonstrated that encapsulated rapamycin keeps its biological effect and efficiently inhibits mTOR phosphorylation. LNC-rapa were more cytotoxics than rapamycin alone but, in association with 8Gy radiation, no synergistic effect were observed. This result could be explained by the complexity of the PI3k/Akt/mTOR in GB as demonstrated by activation of phosphorylated Akt with mTOR inhibition and dependence from oxic status.

## Materials and Methods

### Materials

Lipoïd® S75-3 (soybean lecithin at 69% of phosphatidylcholine) and Solutol® HS15 (a mixture of polyethylene glycol 660 and polyethylene glycol 660 hydroxystearate) were kindly provided by LipoïdGmbh (Ludwigshafen, Germany) and BASH (Ludwigshafen, Germany), respectively. NaCl and DMSO were provided by Sigma Aldrich (St-Quentin, Fallavier, France). Deionized water was obtained from a Milli-Q plus system (Millipore, Paris, France). Lipophilic Labrafac® CC (caprylic-capric acid triglycerides) was provided by Gattefosse S.A. (Saint-Priest, France). Rapamycin was purchased from Interchim (Montluçon, France).Captex® 8000 (Triglyceride of caprylic acid), Transcutol® HP (Diethylene glycol monoethyl ether), and Miglyol® 812 (caprylic/capric triglyceride) were purchased, respectively, from Abitec (Janesville, WI, USA), Gattefosse S.A. (Saint-Priest, France) and Sasol Germany GmbH (Marl, Germany).

### Reagents and Antibodies

Rapamycin was dissolved in DMSO. The final concentration of DMSO in the culture medium did not exceed 0.2%. Anti-phospho-mTOR (ab109268, diluted 1:2,000) and anti-HIF-1α (ab51608, diluted 1:2,000) were from Abcam (Cambridge, UK). Anti-phospho-Akt (#4058, diluted 1:1,000) was from Cell Signaling Technology (Beverly, MA, USA) and anti-HSC70 (sc7298, diluted 1:10,000) was from Santa Cruz biotechnology (Dallas, TX, USA). Peroxidase-conjugated anti-mouse (#32430, diluted 1:2,000) and anti-rabbit (#32460, diluted 1:2,000) secondary antibody were from ThermoScientific (Waltham, MA, USA). Lysis buffer: [50 mM Hepes (pH 7.5), 150 mM sodium chloride, 1 mM EDTA (pH 8), 2.5 mM EGTA (pH 7.4), 0.1% Tween 20, 10% glycerol, 0.1 mM sodium orthovanadate, 1 mM sodium fluoride and 10 mM β-glycerophosphate] plus Protease inhibitor cocktail (#539134 Calbiochem, Darmstadt, Germany), PMSF and Phosphatase inhibitor Cocktail Set II (#524636 Calbiochem).

### Solubility Assays

Rapamycin solubility assays were performed in different oils: Captex® 8000, Labrafac® CC and Miglyol® 812. Five microgram of rapamycine were dissolved in 250 mg of oil and kept under magnetic stirring during 3 h at room temperature (RT) or at 90°C. Rapamycin concentration was determined by reverse-phase high-performance liquid chromatography (RP-HPLC) after 24 h settling at 4°C, using μBondapack C18 column (Waters Corporation, Milford, MA) with an ultraviolet detector at 278 nm. The mixture of 90% acetonitrile and 10% water (v/v) was used as a mobile phase, and delivered at a flow rate of 2.0 mL/min. The injection volume was 10 μL and the retention time was about 2.3 min.

For spectral analysis of the stability of rapamycin in Labrafac®, rapamycin was solubilized at 1 mg/mL in Labrafac® under magnetic stirring before being submitted to 3 to 6 short cycles of heating (70°C for <1 min) and cooling (RT) or incubated for 1 to 3 h at 70°C in Labrafac. Spectral analysis was then made by use of the CLARIOstar microplate reader (BMG Labtech, Champigny-sur-Marne, France).

### Formulation and Physico-Chemical Characterization of Empty (LNCs) and Rapamycin-Loaded Lipid Nanocapsules (LNC-rapa)

LNCs were prepared according to a phase-inversion process adapted from Heurtault et al. (2002). This process involves the formation of an oil/water microemulsion containing an oily/fatty phase (triglycerides: Labrafac® WL 1349), a non-ionic hydrophilic surfactant (polyethylene glycol hydroxystearate: Solutol® HS15), and a lipophilic surfactant (lecithin: Lipoïd® S75-3). Briefly, 21 mg of Lipoïd® S75-3, 138 mg of Solutol® HS15, 345 mg of Labrafac®, 104 mg of NaCl and 898 mg of deionized water were mixed by magnetic stirring. 5 mg of rapamycin were added to other reagents for a final concentration of 1 mg/mL. Three cycles of progressive heating and cooling between 30 and 70°C were then carried out and followed by an irreversible shock, induced by addition of 3.6 mL of 0°C deionized water. Afterwards, slow magnetic stirring was applied to the suspension for 5 min. LNCs were filtered through a Minisart® 0.2 μm filter (Sartorius, Goettingen, Germany) and kept at 4°C. The average diameter and polydispersity index were determined using Malvern Zetasizer® Nano Serie DTS 1060 (Malvern instruments S.A., Worcestershire, UK).

Encapsulation of drug: For determination of drug encapsulation yield, three samples of filtrate were prepared by dissolution of an exact quantity of LNC dispersion in a 96/4 (v/v) methanol/tetrahydrofurane solution. Free rapamycin (non-soluble) was removed by the filtration performed through the Minisart® 0.2 μm filter and its concentration measured by spectrophotometry at 289 nm. Quantification was achieved by comparison between observed peak area ratios of rapamycin of the samples and a calibration curve performed using the same conditions. Samples were performed in triplicate and the loading capacity (LC) was calculated using the following equation:

(1) $$\begin{array}{l} \text{Drug content }\left(\text{wt}\%\right)=\frac{\begin{array}{c} \text{mass of encapsulated}\\ \text{ drug} \end{array}}{\begin{array}{c} \text{mass of encapsulated drug }+\text{mass of }\\ \text{LNC excipients} \end{array}}\\ \;\times 100 \end{array}$$

The encapsulation efficiency (EE) of rapamycin was calculated using the Equation (2):

(2) $$\begin{array}{l} \text{Encapsulation efficiency }\left(\text{wt}\%\right)=\frac{\text{mass of encapsulated drug}}{mass\;of\;initial\;drug}\\ \;\times 100 \end{array}$$

For electrical conductivity measurements, an electrical conductivity meter (Cond 330i/SET, WTW, Germany) was used in non-linear temperature compensation mode according to EN 27888. The conductivity variations were followed as a function of temperature to determine the emulsion inversion zone.

### Cell Culture and Exposure to Hypoxia

Human malignant glioma cell lines U87MG were purchased from American Tissue Culture Collection (Rockville, MD). Tumor cells were cultured in Dulbecco's modified Eagle's medium 4.5 g/L glucose and L-glutamine (DMEM, Lonza, Verviers, Belgium) supplemented with 10% of heat-inactivated fetal bovine serum (FBS, Lonza) and 1% antibiotics suspension (10 units/mL of penicillin, 10 mg/mL streptomycin and 25 μg/mL amphotericin B, Sigma-Aldrich, Saint-Louis, MO, USA). Tumor cells were incubated at 37°C in 5% CO_2_ and 21% (normoxia) or 0.4% O_2_ (hypoxia). Hypoxia conditions were obtained by use of an InVivO_2_ 400 SCI-tive hypoxia workstation (Ruskinn Technology, Ltd., Leeds, UK).

### Irradiation Procedure

Irradiation was performed with the CP-160 cabinet x-ray system (Faxitron, Edimex, Le Plessis Grammoire, Angers, France) which delivers a dose of 1,5 grays by min. Irradiation was performed during 5.33 min in order to reach the dose of 8 grays. Irradiation was performed with cells covered. Depending on the condition considered, the cells were placed throughout the experiment in a conventional 21% O_2_ incubator at 37°C/5% CO_2_ (normoxia) or 0.4% O_2_ (hypoxia) at 37°C/5% CO_2_ in an InVivO2 400 SCI-tive hypoxia workstation (Ruskinn); they are only placed in an isolated flask for the duration of the irradiations.

### Cytotoxicity Evaluation

Two assays were performed to determine the cytotoxicity effect of LNC-rapa on the glioblastoma cell line U87MG:MTS (3-(4,5-dimethylthiazol-2-yl)-5-(3-carboxymethoxyphenyl)-2-(4-sulfophenyl)-2H-tetrazolium) (Promega, Charbonnières, France) and clonogenic assay by crystal violet coloration (Sigma-Aldrich).

For the MTS assay, U87MG cells (5 × 10^4^ cells/mL) harvested in the exponential growth phase were seeded in a 24-well plate in DMEM medium with 10% FBS, in humidified atmosphere (5% CO_2_) at 37°C. Once the cells incubated in the exponential growth phase, serum-contained medium was removed and replaced by serum-deprived DMEM supplemented with 1% N1 supplement (Sigma-Aldrich). Free rapamycin dissolved in DMSO (1/10,000, non-toxic) was applied at various concentrations (0.04; 0.2; 1; 5; 10; 20; 100; 200 μM) for 4 h. 8Gy radiation was performed 6 h after the onset of initial treatment by Faxitron CP-160 (Faxitron X-rays, Lincolnshire, UK). Medium was changed every day. Forty-eight hours following the treatment, MTS reagent was diluted (1:5) in U87MG cell medium and incubated for 2 h at 37°C. The absorbance was measured at 492 nm using Multiskan® microplate spectrophotometer (Thermo Scientific).

For the clonogenic assay, U87MG cells (10^3^ cells/mL) harvested in the exponential growth phase were seeded in a 6-well plate in DMEM medium with 10% FBS, in humidified atmosphere (5% CO_2_) at 37°C. Once the cells incubated in the exponential growth phase, serum-contained medium was removed and replaced by serum-deprived DMEM supplemented with 1 % N1 supplement. Cells were treated for 4 h with rapamycin, LNC-rapa at 1 μM (IC50 LNC-rapa at 21% O_2_ corresponding to a 1/1,000 dilution from initial suspension) and with empty LNCs at the same dilution than LNC-rapa. 8Gy radiation was performed 6 h after the treatment by Faxitron CP-160. Ten days after treatment, colonies were colorized by crystal violet and their number was evaluated with ImageJ Software version 1.43.

Depending on the condition considered, the cells were placed throughout the experiment in a conventional 21% O_2_ incubator at 37°C/5% CO_2_ (normoxia) or 0.4% O_2_ (hypoxia) at 37°C/5% CO_2_ in an InVivO2 400 SCI-tive hypoxia workstation (Ruskinn); they are only placed in isolated flasks for the duration of the irradiations.

### Western Blotting

U87MG cells (2.4 × 10^5^ cells/mL) harvested in the exponential growth phase were seeded in dishes in DMEM medium with 10% FBS, at 37°C in humidified atmosphere containing 5% CO_2_ and 21 or 0.4% O_2_. Once the cells incubated in the exponential growth phase, serum-contained medium was removed and replaced by serum-deprived DMEM supplemented with 1% N1 supplement. Cells were treated with rapamycin, LNC-rapa at 1 μM and with empty LNCs at the same dilution than LNC-rapa. 8Gy radiation was performed 6 h after the treatment by Faxitron CP-160 (cf. section Irradiation Procedure).

Sixteen hours after rapamycin initial treatment (untreated, rapamycin, LNC, LNC-rapa), soluble proteins for immunobloting were harvested from tumor cells lysed in 300 μL lysis buffer on ice. Cells were scrapped and lysed by sonication for 10 s.

Equal amounts of protein from each sample, estimated by the Bio-Rad Protein Assay (Richmond, CA), were separated by electrophoresis through a 4–20% SDS-polyacrylamide gel (Mini-protean® TGX™ Ge, BioRad), transferred to PVDF membranes (AmershamHybond, GE Healthcare, Buckinghamshire, UK) and blocked with 4% non-fat dry milk in 1X TBS plus 0.1% Tween 20 at RT for 1 h. The membranes were washed and incubated with a primary antibody diluted in 2% BSA in 1X TBS plus 0.1% Tween 20 overnight at 4°C. The membranes were then washed and incubated again for 1 h at RT with peroxidase-conjugated anti-rabbit or anti-mouse secondary antibody. The bound antibody was detected using the enhanced chemiluminescence reagent kit SuperSignal West Femto (Thermo Scientific, Waltham, MA, USA) and read with a bioluminescence detector Image Quant Las 4000 (GE Healthcare, USA).

### Statistical Analysis

Three independent biological replicates were performed for all experiments described in this manuscript. Statistical analyses were performed with R software using two-way *analysis* of variance (ANOVA) test. Differences were considered significant if the *p*-value was ≤ 0.05.

## Results

### Formulation and Physicochemical Characterization of Rapamycin-Loaded LNCs

As a lipophilic molecule with logP = 4.3, rapamycin can be encapsulated in the lipophilic core of lipid nanocapsules. The formulation of LNCs *via* a phase-inversion process described by Heurtault et al. (2002) involves three cycles of heating/cooling between 60 and 90°C. However, rapamycin degrades at higher temperatures as observed during the solubility assay. Three different oils were tested for the dissolution of rapamycin at room temperature (RT) and at 90°C: Captex® 8000, Miglyol® 812 and Labrafac®. Rapamycin concentration in the supernatant was determined by HPLC and the results are summarized in Table 1. At 90°C, rapamycin is completely degraded whatever the oil used. At RT, rapamycin has a comparable solubility in all three oils.

**Table 1:** Rapamycin solubility in different oils at RT and at 90°C.

**Oil**	**Temperature**	**Initial rapamycin (mg/mL)**	**Dissolved rapamycin (mg/mL)**	**Dissolution rate (%)**
Captex® 8000	RT	19.0	1.8	9.5
	90°C	16.8	0	0
Miglyol® 812	RT	19.2	1.4	7.3
	90°C	17.1	0	0
Labrafac®	RT	24.0	1.5	6.3
	90°C	21.1	0	0

**Figure 1:**
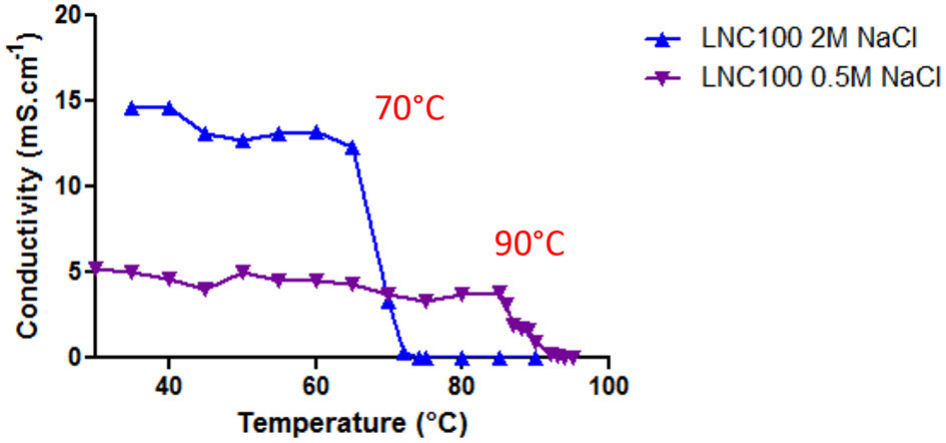
Micro-emulsion conductivity. Micro-emulsion conductivity during cycles of heating/cooling for LNCs formulation performed with 2 M or 0.5 M NaCl aqueous solution.

Finally, Labrafac®, pharmaceutically acceptable and in which the stability of rapamycin is confirmed during short cycles of heating and cooling at 70°C (Supplementary Figure 1), was used for the formulation of empty and rapamycin-loaded LNCs. Hence, a lower temperature (70°C) was employed in order to avoid rapamycin decomposition. To decrease the phase inversion temperature from 90 to 70°C, we increased the concentration of NaCl aqueous solution. Electrical conductivity of the micro-emulsion was measured as a function of temperature for the “classical formulation” with 0.5 M NaCl and for the formulation with 2 M NaCl (Figure 1). A steady state at a high conductivity value indicates that the continuous phase of the emulsion is water, whereas conductivity close to zero means that the continuous phase is oil. The region where the conductivity gradually changes with temperature represents the phase inversion from oil-in-water emulsion to water-in-oil emulsion. Figure 1 shows that the phase inversion occurs at lower temperature (70°C) when 2 M NaCl aqueous solution is used as compared to 0.5 M NaCl solution (90°C). Thus, increasing NaCl concentration allows us to perform rapamycin encapsulation in non-degrading temperature range between 30 and 70°C.

Empty and rapamycin-loaded LNCs were characterized in terms of their average size and zeta potential. These values are presented in Table 2. LNC-rapa have an average size of 112.6 ± 8.4 nm with a polydispersity index (PDI) of 0.044 ± 0.011. The zeta potential is of −5.5 ± 0.4 mV. Rapamycin encapsulation efficiency and loading capacity were determined using the equations 1 and 2, these values are also reported in Table 2. The encapsulation efficiency is of 68.8 ± 7.1 wt% thus representing a loading capacity of the nanoparticle of 0.6 ± 0.1 wt%. This encapsulation efficiency rate was considered in the calculation of rapamycin concentration in biological assays. Insofar as low temperature-made LNC can exhibit fluctuations in their long-term stability with regard to preservation methods not yet fully elucidated, the LNCs used throughout of this work were prepared extemporaneously (Supplementary Table 1).

**Table 2:** Physicochemical parameters of LNC-rapa.

	**Inversion phase (^**°**^C)**	**Size (nm)**	**PdI**	**Zeta-potential (mV)**	**Encapsulation efficiency (% w/w)**	**Loading capacity (%w/w)**
LNC-rapa	70	112.6 ± 8.4	0.04 ± 0.01	−5.5 ± 0.5	68.8 ± 7,1	0.6 ± 0.1
LNC	90	92.3 ± 2.6	0.05 ± 0.02	−8.6 ± 0.6	0	0

*Average particle size, PDI, zeta potential, encapsulation efficiency (EE) and loading capacity (LC) of empty and rapamycin-loaded LNCs*.

### Effect of Rapamycin-Loaded LNCs (LNC-rapa) on mTOR Phosphorylation in U87MG Cells Depending on Oxic Condition and Exposure to Radiation Treatment

Rapamycin binds FKBP12 and the complex FKBP12/rapamycin inhibits mTOR phosphorylation that leads to 4E-BP1 dephosphorylation and inhibition of translation. To check if rapamycin encapsulated within LNCs keeps its biological proprieties, human U87MG glioblastoma cells, that are PTEN negative and thus overactivate Akt/mTOR signals, were treated with empty LNCs, LNC-rapa and free rapamycin dissolved in DMSO. The cells were cultured in serum-free medium in atmosphere containing either 21% O_2_ or 0.4% O_2_.

As cytotoxicity assay performed by MTS with free rapamycin demonstrated a toxic effect only at high concentrations, with more impact in normoxia than in hypoxia (IC50 of 20.54 μM at 21% pO_2_ and 34.65 μM at 21% pO_2_ and 0.4% pO_2_, respectively, Supplementary Figure 2), the choice to use a relevant far much lower concentration while using the LNC nanocarrier was made. Hence a concentration of 1 μM (corresponding to the IC50 LNC-rapa at 21% O_2_ and to a 1/1,000 dilution from the initial suspension while using LNC) was applied all throughout the work.

**Figure 2:**
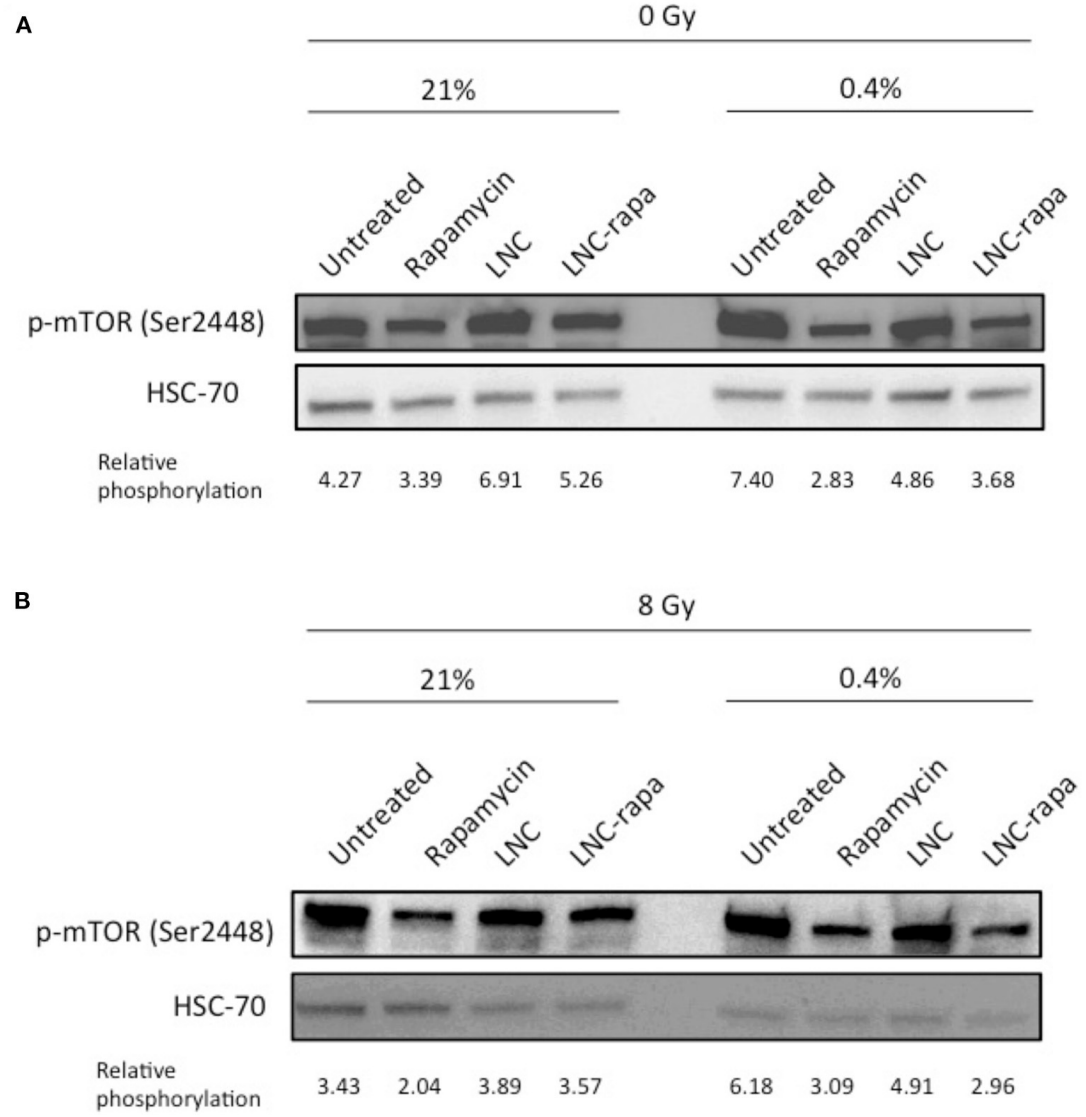
Effects of LNC-rapa assessed on mTOR phosphorylation in U87MG cells depending on oxic condition and exposure to radiation treatment. U87MG cells were treated with rapamycin, empty LNCs (LNC) or rapamycin-loaded LNCs (LNC-rapa), and maintained at two oxygenation conditions: 21 and 0.4% O_2_ before proceeding to western blot analysis. Relative phosphorylation of one representative experiment was determined by volumetric ratio of p-mTOR/HSC70. **(A)** 0Gy. **(B)** 8Gy irradiation.

Western blot analysis was performed and relative phosphorylation was determined by volumetric ratio of p-mTOR/HSC70. The results presented in Figure 2A indicate that rapamycin-encapsulated within LNCs effectively inhibits mTOR phosphorylation (Ser2448) with modalities much more effective in hypoxia than in normoxia.

This observation is consistent with the one made by Brugarolas and coworkers who notably showed that hypoxia induced mTOR inhibition through TSC1/TSC2 tumor suppressor complex and the hypoxia-inducible gene REDD1/RTP801. They demonstrated that in contrast to energy depletion, mTOR inhibition by hypoxia does not require AMPK or LKB1 but depend on increased expression of the hypoxia inducible *REDD1* gene. They also showed that down-regulation of S6K, an mTOR target, phophorylation by Redd1 requires Tsc2 and Redd1 probably acts up-stream of the Tsc1/Tsc2 complex to down-regulate mTOR function in response to hypoxia (Brugarolas et al., 2004). Thus, at 0.4% oxygenation, mTOR is inhibited by rapamycin and hypoxia, with loaded-LNCs also exerting a higher effect in these conditions (Figure 2A).

As various synergies have been tested and since the conventional treatment of glioblastoma involves beam radiation, the impact of LNC-rapa on mTOR phosphorylation in U87MG cells was also tested after exposure to 8Gy irradiation. Similar results to the non-irradiated condition are obtained (Figure 2B).

### Effect of LNC-rapa on U87MG Cell Growth Depending on Oxic Condition and Exposure to Radiation Treatment

**Figure 3:**
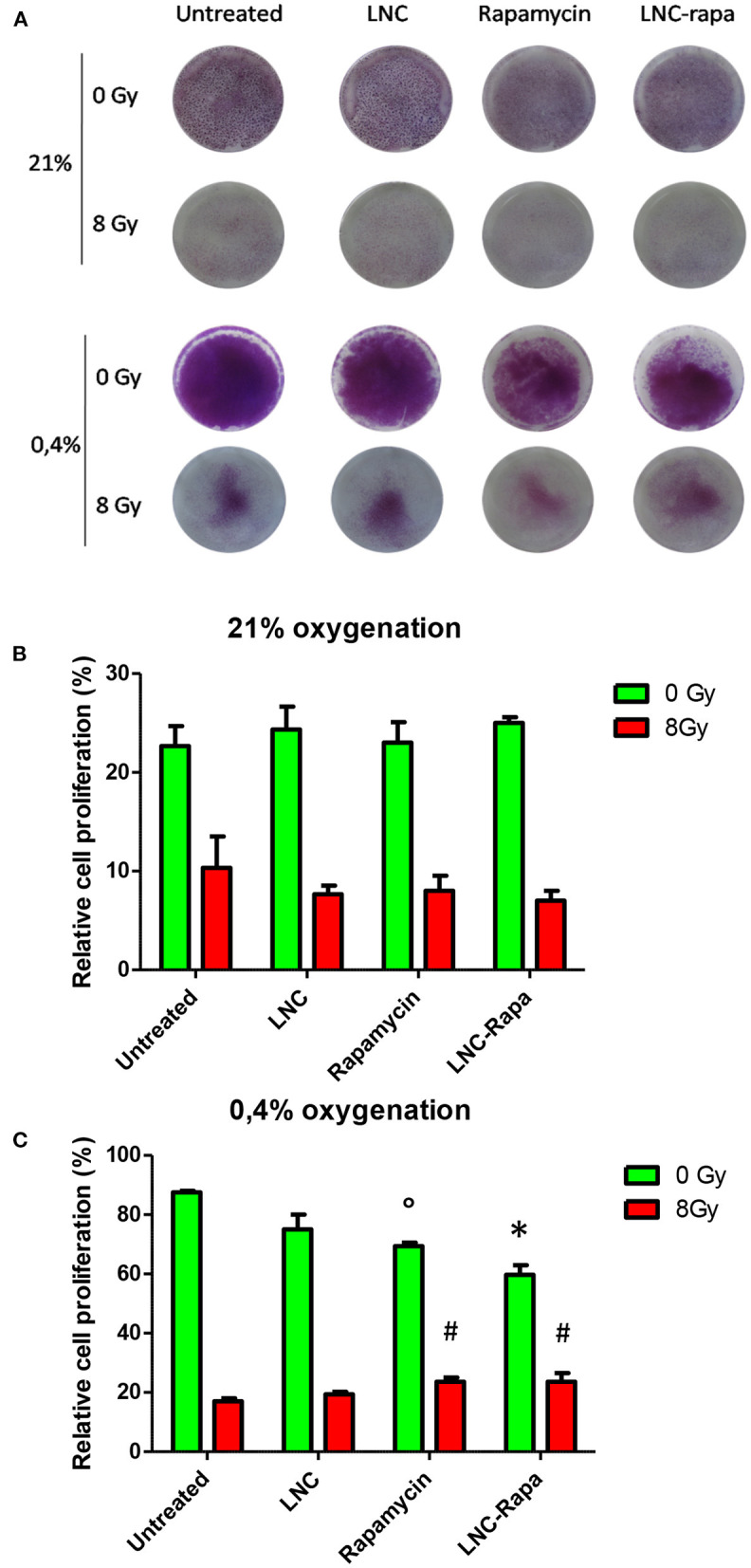
Effects of LNC-rapa assessed by clonogenic assay on U87MG cell growth depending on oxic condition and exposure to radiation treatment. **(A)** Photography of 6-wells plates containing U87MG cells treated with LNCs, rapamycin or LNC-rapa, radiated at 0Gy (top row) or 8Gy (bottom row) at 21 and 0.4% p0_2_ and stained with crystal violet. **(B,C)** Cell survival was determined by measuring crystal violet staining of wells exposed to 0 and 8Gy at 21% pO_2_ **(B)** or 0.4% pO2 **(C)**. Data show the average values from a combination of three independent experiments and error bars display the standard deviation. Two-way ANOVA test was performed between LNC-rapa condition compared to LNC condition (*p ≤ 0.05) or between rapamycin treatment condition and untreated control condition (°*p* ≤ 0.05) or between rapamycin treatment condition and untreated condition (^#^*p* ≤ 0.05).

To determine the effect of rapamycin encapsulated within LNCs on cancer cell survival and growth depending on the oxygen status and exposure to radiation treatment, a clonogenic assay was performed. Hence, U87MG cells were grown under two oxygenation conditions (21 or 0.4% O_2_) and treaded with either empty LNCs, LNC-rapa or free rapamycin at 1 μM before being exposed, 6 h later, to 0 or 8Gy irradiation. They were then maintained in culture for 10 days and colorized by crystal violet (Figure 3A). Under all the conditions tested, a very clear effect of the irradiations, in normoxia (21% pO_2_) as in hypoxia (0.4% O_2_), was observed (Figures 3B,C). Rapamycin, nanovectorized or as free, exerts only moderate effects, however significant at 0.4% O_2_, demonstrating the similarity of action of encapsulated LNC-rapa vs. the free form (Figure 3C). Interestingly, rapamycin and LNC-rapa do not exert any synergistic effect related to radiation treatment and even slight but significant inhibitory effects impacted radiation efficacy at 0.4% O_2_ (Figure 3C).

### Activation of Alternative Signaling Pathways in Response to Exposure to LNC-rapa in U87MG

**Figure 4:**
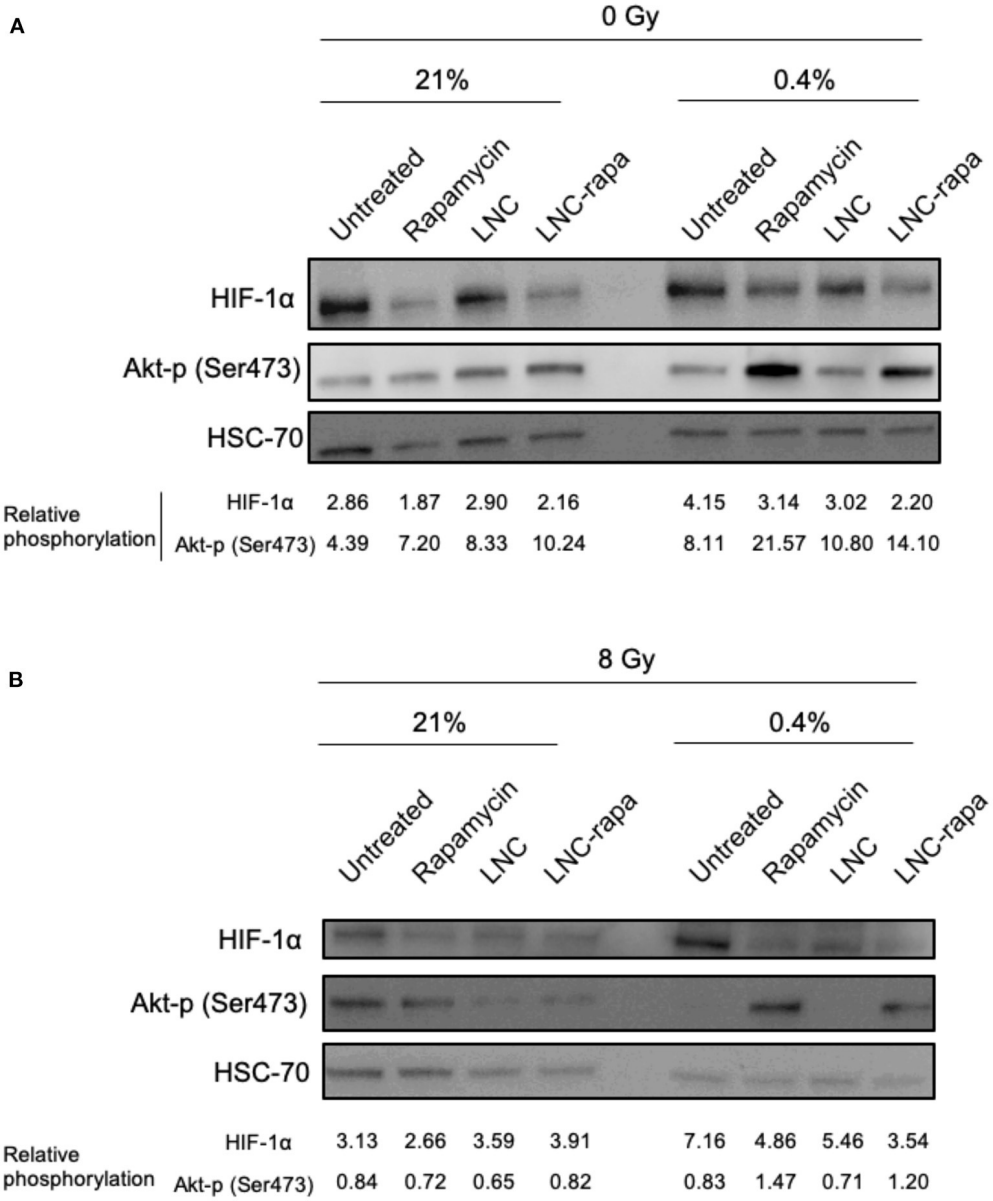
Activation of alternative signaling pathways in response to exposure to LNC-rapa in U87MG. **(A,B)** U87MG cells were treated with free rapamycin, empty LNCs or LNC-rapa, radiated at 0Gy **(A)** or 8Gy **(B)** and maintained at two oxygenation conditions: 21 and 0.4% before proceeding to western blot analysis. Relative phosphorylation of one representative experiment was measured by volumetry ratio of p-mTOR/HSC70.

The observed duality of the effects of rapamycin and LNC-rapa associating a strong inhibition of mTOR phosphorylation to a moderated cytotoxic effect whatever the environmental conditions used (low/high oxygen or irradiating) led us to focus on the mechanisms that control the PI3K/Akt/ mTOR pathway. Since HIF exerts negative feedback on mTOR (Brugarolas et al., 2004) and mTORC2 complex also exerts feedback control while capable to phosphorylate Akt (Sarbassov et al., 2005; O'Reilly et al., 2006), HIF-1α protein expression and phosphorylation of Akt on Ser473 (Akt-p) were evaluated. Western Blot presented in Figure 4A shows that HIF-1α protein expression is reduced when cells are treated with free rapamycin and LNC-rapa whatever oxygenation condition considered. Inversely, these treatments enhance Akt-p protein level. Figure 4B shows that at 8Gy, Akt-p protein expression is reduced related to HSC70 in comparison with the 0Gy control condition. Again the down regulation of HIF-1α protein expression by free rapamycin and LNC-rapa is observed concomitantly with the induction of phosphorylation of Akt, thus emphasizing the possible double edge sword impact of LNC-rapa due to the multiplicity of signals downstream mTOR inhibition.

## Discussion

This work demonstrated that a new safe formulation of rapamycin encapsulated in lipid nanocapsules at low temperature and without the use of organic solvent, allows keeping its activity while specifically inhibiting mTOR phosphorylation. These observations also established that the mechanism of action of rapamycin-loaded LNCs, to some extent like free rapamycin, involve distinct modalities of responses at 0.4 vs. 21% oxygenation. Indeed, protein expression analysis shows that, if mTOR phosphorylation inhibition is higher at 0.4% O_2_, the up-stream effector of PI3k/Akt/mTOR pathway, Akt phosphorylation, is higher too. Furthermore, free rapamycin and LNC-rapa inhibit HIF-1α expression at 21% O_2_ and to a lesser extent at 0.4% O_2_. This difference is linked to HIF-1α stabilization under hypoxia.

### LNC-rapa as a New Safe Nanocarrier of Rapamycin

In the present study, we developed lipid nanocapsules capable to efficiently encapsulate rapamycin with yield close to 70%. The formulation was done between 30 and 70°C, a temperature range that protects rapamycin from thermal degradation. Capable to cope with poor water solubility of rapamycin and bioavailability due to their capability to effectively reached intracellular cell compartment (Paillard et al., 2010), rapamycin-loaded LNCs keep rapamycin biological proprieties with an effective inhibition of mTOR phosphorylation. Although this tool fulfills its role as a vector, it does not strengthen the activity of rapamycin or one of its selective aspects in our *in vitro* model tested as well as through multiple conditions (8Gy irradiation, 0.4% hypoxia, 21% normoxia).

In the plethora of new rapamycin nanovector formulations currently available, the loading capacities of each of them, their application methods and loco-regional bioavailability should make it possible to resolve the problem of efficiency and possibly synergy with conventional treatments. Thus a loading capacity of 0.6% for LNC-rapa remains low compared to other systems such as polysorbate 80-coated PLGA nanoparticles (Escalona-Rayo et al., 2019), lipid-polyaniline nanoparticles (Wang J. P. et al., 2016) or PEO/PDLLA electrospun nanofibers (Wang B. L. et al., 2016). Comparative studies in particular *in vivo* should make it possible to understand the rationale which makes one of these vectors an appropriate tool or not.

Forrest and coworkers have developed poly(ethylene glycol)-b-poly(ε-caprolactone) (PEG-PCL) micelles loaded with rapamycin and showed that this drug was efficiently loaded within PEG-PCL up to 10 wt% (more than 1 mg/mL) (Forrest et al., 2006). Other group also demonstrated that rapamycin encapsulation within poly(ethylene glycol)-Block-poly(2-methyl-2-benzoxycarbonyl-propylene carbonate) (PEG-b-PBC) micelles reduced its toxicity (Lu et al., 2011). Shi et al. (2013) developed elastin-based protein polymer nanoparticles loaded with rapamycin and decorated with its ligand FKBP. They showed that these objects slowed down the drug release as compared to non-decorated nanoparticles. Moreover, rapamycin elastin-like polypeptide nanoparticles decreased the gross toxicity and enhanced the anti-cancer activity on human breast cancer mice model (Dhandhukia et al., 2017a,b; Peddi et al., 2020). Finally, Tyler and coworkers incorporated rapamycin into biodegradable caprolactone-glycolide (35:65) polymer beads (Tyler et al., 2011). *In vitro*, rapamycin was cytotoxic toward 9L cells (rat glioma cells), causing growth inhibition at a concentration of 0.01 μg/mL. No *in vivo* toxicity was observed at 0.3, 3, and 30% loading doses implanted intracranially. Animals treated with the highest dose of rapamycin beads (30%) consistently demonstrated significantly longer survival duration than the control and placebo groups. They also showed that radiation therapy in addition to the simultaneous treatment with 30% rapamycin beads led to significantly longer survival duration than each therapy alone.

### Vectorized Rapamycin: A Double-Edge Sword “Interactor” in Cancer Cells

**Figure 5:**
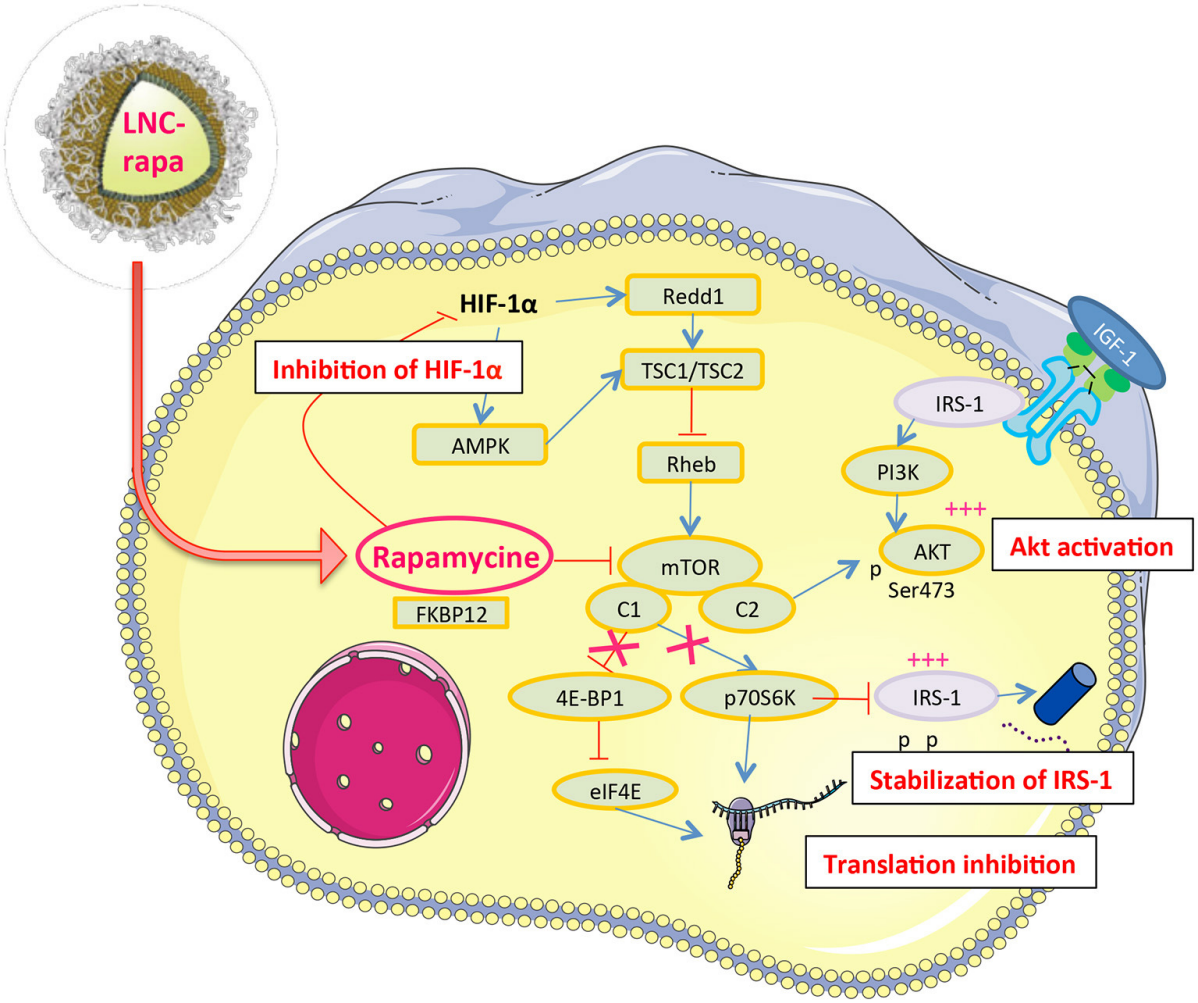
Rapamycin a likely double-edge sword molecular interactor in U87MG glioblastoma cells. Nanovectorized rapamycin (LNC-rapa) or rapamycin as free, solubilized in DMSO, inhibits the stabilization and transcriptional activity of HIF1α which in turn inhibits the TSC1/TSC2 inhibitor and thus promotes the activation of mTOR. On the other hand, rapamycin inhibits mTORC1 but not mTORC2, which in turn induces Akt phosphrylation. Finally, by inhibiting mTORC1, rapamycin lifts the inhibition exerted by p70s6k on IRS-1, which stabilizes this protein and induces Akt phosphorylation *via* the IGF-1 receptor.

The result we obtained on mTOR phosphorylation by rapamycin and LNC-rapa associated with of HIF-1α down regulation and Akt phosphorylation can be linked to the observation made by Hudson et al. who reported that rapamycin inhibits both stabilization of HIF-1α and the transcriptional activity of HIF-1 in hypoxic cancer cells and mTOR dependent signals stimulate HIF-1α accumulation and HIF-1 mediated transcription in cells exposed to hypoxia or hypoxia-mimetic agent (Zhong et al., 2000). Rapamycin-sensitive functions of mTOR are not essential for the accumulation of HIF-1α but are needed for maximal expression of this protein, as well as for optimal HIF-1-dependent gene expression under hypoxic conditions. The notion that mTOR is a nutrient sensor may be particularly relevant to HIF-1 function, since decrease oxygen tensions are almost inevitable accompanied by limited supplies of glucose and amino-acids in mammalian tissues (Hudson et al., 2002). *In vivo*, rapamycin enhance thrombosis and also an increase in the hypoxic zone (Weppler et al., 2007). Hypoxia causes activation of the TSC1/2 complex, which functions to inhibit mTOR. This can occur both *via* induction of the HIF-dependent gene REDD1, and/or through activation of AMPK (Brugarolas et al., 2004; Liu et al., 2006). Rapamycin may be less effective in hypoxic regions of tumors since mTOR may already be at least partially inactivated by TSC. Thus the amount of hypoxia present at the start of treatment may play a part in determining sensitivity to rapamycin *in vivo* (Weppler et al., 2007) (Figure 5).

The higher Akt phosphorylation at 0.4% could also explain that cells are less sensitive to rapamycin than at 21%. Indeed, U87MG cell line is PTEN *null* that drives to a constitutive activation of the PI3K/Akt/mTOR pathway and could explains its radioresistance. Thus, mTOR inhibition could restore radiosensitivity but our results show that maximal cytotoxic effect was observed with 8Gy radiation and rapamycin or LNC-rapa were not sufficient to improve the cytotoxicity at the concentration of 1 μM. To well-understand this phenomenon, it is important to remind that mTOR exist in two complexes: mTORC1 and mTORC2. mTORC1 contains the mTOR, Raptor, mLST8/GβL, and PRAS40 proteins and controls cell size and protein translation *via* two major substrates, p70S6K and 4E-BP1. Activated S6 kinase causes feedback inhibition of insulin-like growth factor 1 (IGF-1)/insulin signaling by phosphorylating insulin receptor substrate 1 (IRS-1) and causing its degradation (Tremblay et al., 2007). mTORC2 has been shown to phosphorylate Akt at the serine 473 site, which enhances the catalytic activity of Akt already phosphorylated on threonine 308 (Sarbassov et al., 2005). Rapamycin binds to FKBP-12 and this complex then binds to and causes the allosteric inhibition of mTORC1. Rapamycin effectively blocks S6K phosphorylation and also induces Akt S473 phosphorylation and Akt activity (O'Reilly et al., 2006). Physiologic activation of PI3k/Akt signaling is regulated by mTOR-dependent feed-back inhibition of IRS expression and, consequently, IGF-1 receptor (IGF-1R)/insulin receptor signaling (Tremblay et al., 2007). Rapamycin relieves this feedback and induces Akt S473 phosphorylation in an mTORC2-dependent manner, leading to Akt activation, which may attenuate its therapeutic effects (O'Reilly et al., 2006). Furthermore, mTOR inhibitory drug rapamycin up-regulates IRS-1 protein levels and induces Akt phosphorylation that increase IGF—IR/IRS-1/PI3K signaling to Akt (O'Reilly et al., 2006) (Figure 5). In line with this, mTORC2, not inhibited by LNC-rapa, has recently been described as a downstream integrator of metabolic and epigenetic landscape leading to tumor cell survival and cancer durg resistance (Masui et al., 2019, 2020).

In response of those problems, Rodrik-Outmezguine et al. (2011) used a selective ATP-competitive mTOR kinase inhibitor AZD8055. This drug inhibits 4E-BP1 phosphorylation more effectively than rapamycin. It also inhibits mTORC2 and Akt S473 phosphorylation, which leads to Akt T308 dephosphorylation and suppression of Akt activity and downstream signaling. Unfortunately, even though mTORC2 inhibition is potent and persistent, inhibition of Akt T308 and Akt substrate phosphorylation is only transient. Authors demonstrated that this re-induction resulted from hyperactivation of PI3K. In cells in which mTOR kinase inhibitors relieve feedback inhibition of receptor tyrosine kinase, leading to activation of PI3K, the result is a new steady state in which mTORC1 is potently inhibited and Akt is phosphorylated on T308 but not on the S473. This Akt species is activated and able to phosphorylate key substrates in the cells. Induction of PI3K activation depends also from cell directory of activated tyrosine kinase receptors and from active ligands available (Rodrik-Outmezguine et al., 2011).

Alternatively, Kahn and coworkers showed *in vitro* that addition of AZD2014, another mTORC1/mTORC2 inhibitor, to culture media 1 h before irradiation enhanced the radiosensitivity of CD133^+^ and CD15^+^ glioblastoma stem-like cells (Kahn et al., 2014). The combination of AZD2014 and radiation delivered to mice bearing GSC-initiated orthotopic xenografts significantly prolonged survival of these animals as compared to individual treatments.

In parallel, dual PI3K/mTOR inhibitors were developed, notably the NVP-BEZ235. It demonstrated suppression of mTORC1 (S6K1, S6K, and 4E-BP1) and mTORC2 (AKT) downstream components resulting in cell cycle arrest and induced autophagy (Cerniglia et al., 2012). NVP-BEZ235 showed inhibited *in vivo* glioma proliferation and improved anti-tumor effects compared to rapamycin analogs. Mukherjee et al. (2012) showed that NVP-BEZ235 can inhibit DNA repair proteins ATM and DNA-PKC in GB that lead to a radiosensitizing effect. Nevertheless, because of the induction of autophagy that seems to be cytoprotective (Cerniglia et al., 2012), combination therapies with NVP-BEZ235 have been explored. One strategy utilized NVP-BEZ235 with autophagy inhibitor chloroquine to show a synergistic effect in *in vivo* tumor apoptosis (Fan et al., 2010). In line with this, Heinze et al., underlined that under hypoxia and nutrient-poor conditions, second generation mTORC1/C2 inhibitors displayed even stronder cytoprotective effect by reducing oxygen and glucose consumption (Heinzen et al., 2019).

However, those experiences were performed mainly *in vitro* and could yield different results *in vivo*. Indeed, some groups have reported that rapamycin sensitized U87MG xenografts to fractionated radiation therapy. Eshleman and coworkers also showed that there were no radiosensitizing effects of rapamycin on U87MG in the radiation clonogenic survival assays, nevertheless, they observed a great effect in the U87 xenograft and spheroids models (Eshleman et al., 2002). They proposed that other factors could also be important for the sensitizing effect of rapamycin. For example, rapamycin induces significant changes in glucose and nitrogen metabolism, and the starvation-like metabolic state induced by rapamycin could potentially decrease oxygen consumption in solid tumors and improve overall tumor oxygenation (Hardwick et al., 1999). Any decrease in the proportion of radioresistant hypoxic cells should significantly increase the efficacy of radiation. The authors also suggested that rapamycin could inhibit host-dependent processes that contribute to the profound sensitizing effect of rapamycin in xenograft model. Furthermore, rapamycin is a potent inhibitor of endothelial cell proliferation *in vitro*, therefore its systemic administration can inhibit angiogenesis. It reduces VEGF production by tumor cells and the inhibition of VEGF-induced proliferation in endothelial cells (Guba et al., 2002).

In the same way, Weppler et al. (2007) investigated the combination of rapamycin with short course of fractionated radiotherapy to minimize the anti-proliferative effect of rapamycin and thus evaluate its potential to contribute to the direct cytotoxic effect of radiation. They found that rapamycin did not significantly improve radiation response but increased variability in tumor response to radiotherapy, with several individual tumors showing large increases in growth delay. Thus, they underlined the importance to determine the biological factors that mediated this differential response in order to potentially identify patients that may benefit from combination treatment.

## Conclusion

To conclude, rapamycin-loaded lipid nanocapsules for peripheral or loco-regional administration developed in this study represent a new safe nanocarrier of rapamycin capable to convey rapamycin and preserves its biological activity on cancer cells. We showed that activation of a negative feedback following mTOR phosphorylation inhibition is a serious brake on rapamycin cytotoxicity. The first solution could consist of changing rapamycin for dual PI3K/mTOR inhibitors like the NVP-BEZ235 which has demonstrated effectiveness *in vivo* (Cerniglia et al., 2012), or mTORC1/mTORC2 inhibitor is AZD2014 which radiosensitizes glioma (Kahn et al., 2014). Nevertheless, rapamycin radiosensitizer effect have been proved *in vivo* or using fractionated radiation protocol (Eshleman et al., 2002). Moreover, if patients are biologically screened to select the most responsive ones, as underlined by Weppler et al., LNC-rapa can potentially be effective with an adapted radiation protocol.

## Data Availability Statement

All data generated or analyzed during this study are included in this published article (and its Supplementary Information files).

## Author Contributions

DS and EG wrote the manuscript. EG, FH, MC, FB, and FL contributed to the conception, design, and funding of the work. DS and SA contributed to the experiments. DS, NB, LR, AD, and EG contributed to manuscript revisions. All authors read and approved the submitted version.

## Conflict of Interest

The authors declare that the research was conducted in the absence of any commercial or financial relationships that could be construed as a potential conflict of interest.

## Supplementary Material

Supplementary Figure 1 Spectral analysis of rapamycin stability in Labrafac®. **(A)** Labrafac spectra at RT. **(B)** Labrafac spectra after 3 h heating at 70°C. **(C)** Rapamycin spectra after 3 h at RT in Labrafac. **(D)** Rapamycin spectra after six short cycles of heating and cooling (70°C to RT) in Labrafac. **(E)** Rapamycin spectra after 1 h heating at 70°C in Labrafac. **(F)** Rapamycin spectra after 3 h heating at 70°C in Labrafac. **(G)** Spectra of rapamycin, previously dissolved in methanol (MeOH), after three short cycles of heating and cooling (70°C to RT) in Labrafac. **(H)** Spectra of rapamycin, previously dissolved in methanol (MeOH), after 3 h heating at 70°C in Labrafac. Each curve represents one representative analysis of a triplicate.

Supplementary Figure 2 Survival of U87MG cells in response of free-rapamycin treatment assessed by use of MTS assay. **(A)** U87MG cells were treated with free rapamycin at 21% (green curve) and 0.4% (red curve) oxygenation. **(B)** Calculated IC50 at 21 and 0.4% oxygenation following rapamycin treatment.

Supplementary Table 1 Stability of 50 nm blank and rapamycin loaded LNC during storage at different temperatures. Note the modification of size and loss of polydispersity after 7 days storage (boxes highlighted in gray).
