# Supplementary figures and images for: Rapamycin-Loaded Lipid Nanocapsules Induce Selective Inhibition of the mTORC1-Signaling Pathway in Glioblastoma Cells
Source: Front Bioeng Biotechnol. 2021 Feb 25;8:602998. doi: 10.3389/fbioe.2020.602998 (PMC7947795; doi:10.3389/fbioe.2020.602998)

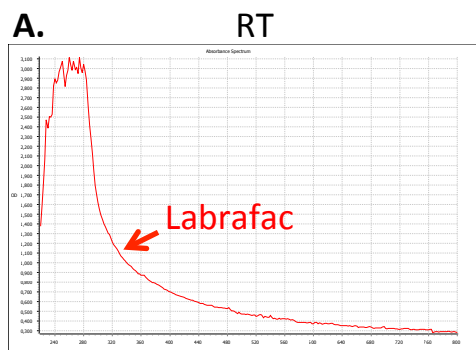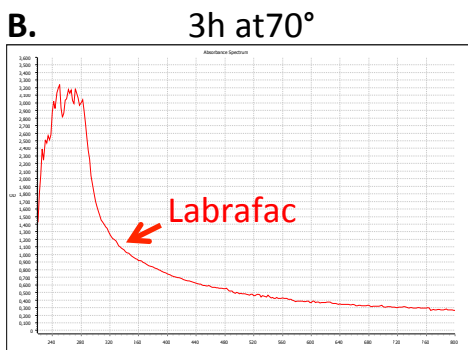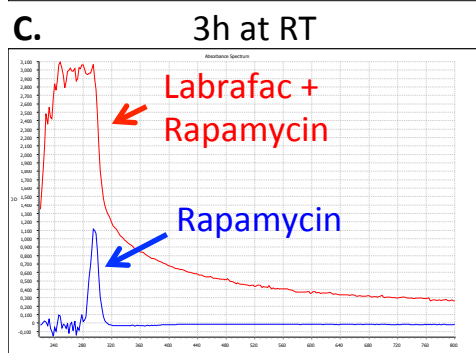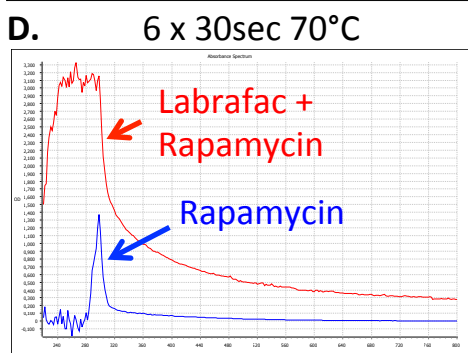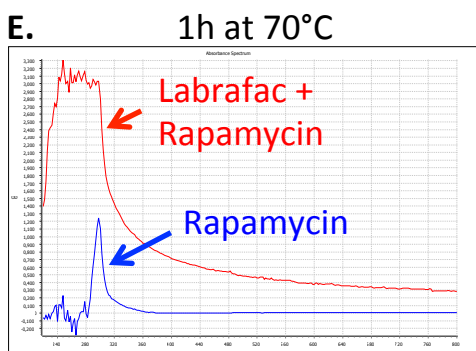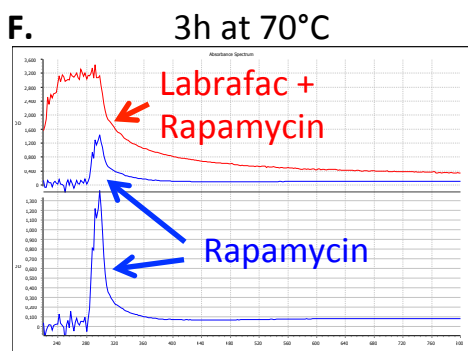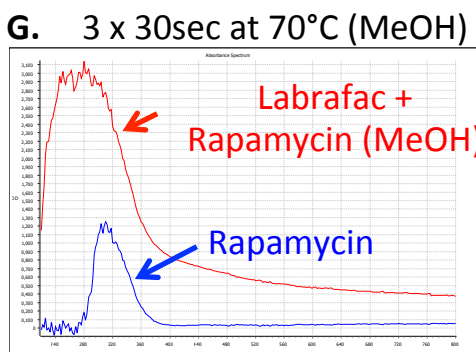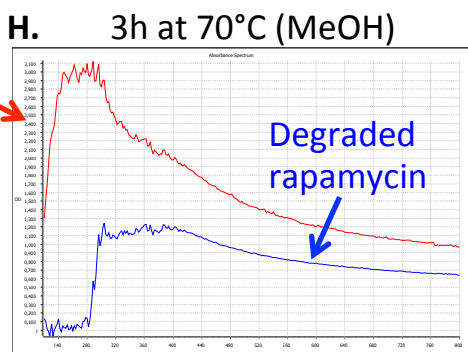

Séhédic et al., Supplemental figure 1

Supplement: Supplementary Figure 1 — Spectral analysis of rapamycin stability in Labrafac®. (A) Labrafac spectra at RT. (B) Labrafac spectra after 3 h heating at 70°C. (C) Rapamycin spectra after 3 h at RT in Labrafac. (D) Rapamycin spectra after six short cycles of heating and cooling (70°C to RT) in Labrafac. (E) Rapamycin spectra after 1 h heating at 70°C in Labrafac. (F) Rapamycin spectra after 3 h heating at 70°C in Labrafac. (G) Spectra of rapamycin, previously dissolved in methanol (MeOH), after three short cycles of heating and cooling (70°C to RT) in Labrafac. (H) Spectra of rapamycin, previously dissolved in methanol (MeOH), after 3 h heating at 70°C in Labrafac. Each curve represents one representative analysis of a triplicate. [file Presentation_1.pdf]

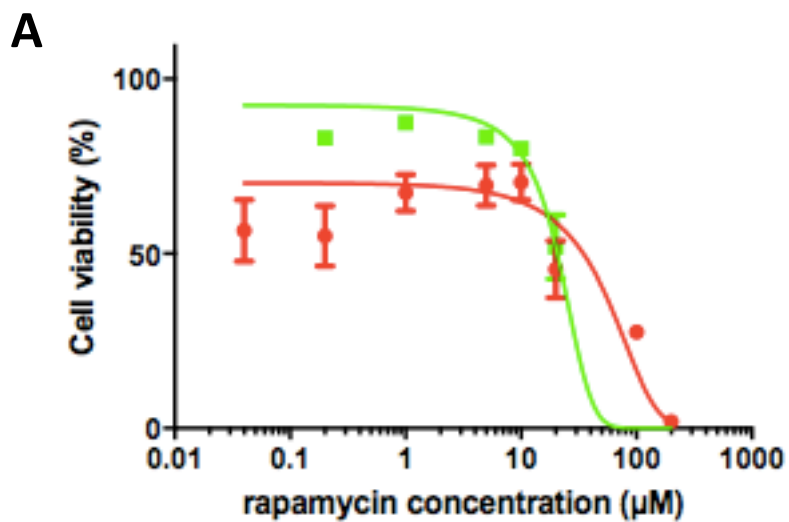

**B**

|                                             | Rapamycin<br>concentration ( $\mu\text{M}$ ) |
|---------------------------------------------|----------------------------------------------|
| IC50 21 % O <sub>2</sub> ( $\mu\text{M}$ )  | 20.54 +/- 2.23                               |
| IC50 0.4 % O <sub>2</sub> ( $\mu\text{M}$ ) | 34.65 +/- 4.31                               |

Supplement: Supplementary Figure 2 — Survival of U87MG cells in response of free-rapamycin treatment assessed by use of MTS assay. (A) U87MG cells were treated with free rapamycin at 21% (green curve) and 0.4% (red curve) oxygenation. (B) Calculated IC50 at 21 and 0.4% oxygenation following rapamycin treatment. [file Presentation_2.pdf]
