# Supplementary material for: Rapamycin-Loaded Lipid Nanocapsules Induce Selective Inhibition of the mTORC1-Signaling Pathway in Glioblastoma Cells
Source: Front Bioeng Biotechnol. 2021 Feb 25;8:602998. doi: 10.3389/fbioe.2020.602998 (PMC7947795; doi:10.3389/fbioe.2020.602998)
Supplement: Supplementary Table 1 — Stability of 50 nm blank and rapamycin loaded LNC during storage at different temperatures. Note the modification of size and loss of polydispersity after 7 days storage (boxes highlighted in gray). [file Table_1.pdf]

Supplemental Table 1: Stability of 50 nm blank and rapamycin loaded LNC during storage at different temperatures.

			50 nm blank-LNC	50 nm rapamycin-LNC
Initial condition		Volume (nm)	48.57 +/- 0.53	46.9 +/- 0.53
		PdI	0.04 +/- 0.01	0.05 +/- 0.02
		Zeta potential (mV)	-5.56 +/- 0.5	-9.83 +/- 0.7
LNC characteristics after 7 days keeping at different temperatures	+4°C	Volume (nm)	51.54 +/- 0.56	79.27 +/- 28.78
		PdI	0.02 +/- 0.01	0.20 +/- 0.09
		Zeta potential (mV)	-4.96 +/- 0.5	-11 +/- 0.8
	-20°C	Volume (nm)	121.40 +/- 9.19	61.25 +/- 1.26
		PdI	0.17 +/- 0.09	0.11 +/- 0.08
		Zeta potential (mV)	-3.99 +/- 0.6	-11.5 +/- 0.7
	-80°C	Volume (nm)	145.20 +/- 1.9	119.8 +/- 5.29
		PdI	0.10 +/- 0.07	0.07 +/- 0.06
		Zeta potential (mV)	-6.04 +/- 0.7	-6.88 +/- 0.5
	-162°C	Volume (nm)	136.60 +/- 0.46	128.3 +/- 3.62
		PdI	0.08 +/- 0.06	0.07 +/- 0.05
		Zeta potential (mV)	-5.17 +/- 0.5	-7.99 +/- 0.6
